# The Key Nutrients in the Mediterranean Diet and Their Effects in Inflammatory Bowel Disease: A Narrative Review

**DOI:** 10.3390/nu16234201

**Published:** 2024-12-05

**Authors:** Sara Deleu, Guia Becherucci, Lihi Godny, Maria Chiara Mentella, Valentina Petito, Franco Scaldaferri

**Affiliations:** 1 CEMAD Translational Research Laboratories, Dipartimento di Scienze Mediche e Chirurgiche, Fondazione Policlinico Universitario Agostino Gemelli IRCCS, 00168 Rome, Italy; 2 Dipartimento di Medicina e Chirurgia Traslazionale, Università Cattolica del Sacro Cuore, 00168 Rome, Italy; 3 Division of Gastroenterology and Nutrition Unit, Rabin Medical Center, Petah-Tikva 49100, Israel; 4 UOC di Nutrizione Clinica, Dipartimento Scienze Mediche e Chirurgiche Addominali ed Endocrino-Metaboliche, Fondazione Policlinico Universitario “A. Gemelli” IRCCS, Università Cattolica del Sacro Cuore, 00168 Rome, Italy

**Keywords:** Mediterranean diet, nutrients, inflammatory bowel disease, IBD

## Abstract

The gut microbiome, a collection of gut microorganisms, is crucial in the development and progression of inflammatory bowel diseases (IBD). Therefore, diet and dietary interventions are promising strategies to shape the gut microbiota for IBD management. Of all the diets studied in the IBD field, the Mediterranean diet has the least restrictive nature, promoting long-term adherence. The Mediterranean diet is rich in plants, with a high daily intake of fruits and vegetables (high in fiber, antioxidants, and vitamins), olive oil, whole grains, legumes, and nuts. It includes the moderate consumption of animal products such as oily fish (rich in mono- and polyunsaturated fatty acids), dairy products, and poultry, with a limited intake of red meat and processed foods. This diet is associated with a decreased risk of chronic diseases, including IBD. However, the mechanisms of specific nutrients behind these effects in the Mediterranean diet remain under investigation. Therefore, in this review, we aim to provide an overview of the nutrients that are abundant in the Mediterranean diet and their effects on IBD, with a main focus on preclinical evidence. While several nutrients like fructo-oligosaccharide, chitosan, plant-derived protein, polyphenols, omega-3 polyunsaturated fatty acids, and resveratrol have shown potential beneficial effects in preclinical models, clinical evidence is often limited. However, understanding the complex interactions between specific nutrients and IBD is essential to developing a tailored, multidisciplinary, and personalized approach for disease management; therefore, further research is required.

## 1. Introduction

The gut microbiome, which is a collection of microorganisms in the human gut, plays a critical role in the pathogenesis of inflammatory bowel disease (IBD), including ulcerative colitis (UC) and Crohn’s disease (CD) [1,2]. Alterations in the composition and function of the microbiome might lead to inflammation and damage to the intestinal wall [3], which can trigger symptoms such as abdominal pain, bloody diarrhea, and weight loss [4]. Diet and dietary interventions have, therefore, emerged as a promising strategy to modulate the gut microbiota in IBD [5,6,7], especially since many nutritional deficiencies (e.g., folate/vitamin B9) have been observed in IBD [8].

The traditional Mediterranean diet is defined as a diet high in fiber and low in saturated/animal fat, with a high intake of fruits, vegetables, whole grains, legumes, nuts, olive oil, and limited consumption of meat and ultra-processed foods [9]. This type of diet has been shown to be associated with the pro- or anti-inflammatory characteristics of the gut microbiome [10] and has been associated with a decreased risk of late-onset CD [11]. In this regard, the consumption of nuts, oily fish, fruit, vegetables, and cereals was associated with a higher abundance of beneficial short-chain fatty acid (SCFA)-producing gut microbiota [10]. Conversely, Western diets high in ultra-processed foods have been associated with negative impacts on IBD risk by affecting the intestinal barrier and altering the composition of the gut microbiota, as detailed by Vissers et al. (2022) [12]. A recent review on the clinical effects of the Mediterranean diet in IBD patients concluded that most clinical evidence supports this diet as an approach for IBD prevention and management [9]. In comparison to other diets investigated for IBD, the Mediterranean diet is less restrictive and may, therefore, allow greater long-term adherence in patients [13,14]. Additionally, the Mediterranean diet is hypothesized to be beneficial by improving the lipid profile, protecting against oxidative stress, inflammation, and platelet aggregation, modifying the hormones and growth factors involved in diseases such as cancer, inhibiting the nutrient-sensing pathways, and finally, modifying the gut microbiota composition [15]. For example, adherence to a Mediterranean diet was shown to improve clinical scores and inflammatory markers in children [16]. Therefore, the latest AGA Clinical Practice Update on diet and nutritional therapies in IBD patients states that unless there is a contraindication, all patients should be advised to follow a Mediterranean diet [8]. Importantly, the traditional Mediterranean diet is more than just a dietary regimen; it is a lifestyle that emphasizes balanced nutrition, regular physical activity, and social connections, which might, together, contribute to its health benefits.

Nevertheless, the exact mechanisms behind the effects of the Mediterranean diet on the gut microbiome in IBD remain under investigation [9]. Therefore, in this review, we aim to provide an overview of the nutrients present in the Mediterranean diet and their effects on IBD, with a main focus on recently obtained preclinical data; when available, clinical evidence is also pinpointed.

## 2. Methods

Data for this review were identified by searches of PubMed and references from relevant articles using the search terms “Mediterranean diet”, “carbohydrates”, “fatty acids”, “olive oil”, “wine”, “protein”, “fat”, “nuts”, “polyphenols”, “spices”, “herbs”, “food additives”, “gut microbiome”, “preclinical”, “in vitro”, “in vivo”, “inflammatory bowel disease” (“IBD”), and any combinations of these terms. Recent articles published in peer-reviewed journals in English between 2019 and 2024 were included, and earlier articles were also considered when highly relevant (>100 citations), e.g., meta-analyses. Very limited clinical evidence is currently available on the effects of nutrients in the Mediterranean diet; therefore, we choose to focus on preclinical data here, although, when available, clinical data are discussed for translational reasons.

## 3. Carbohydrates

Carbohydrates, including inulin, galacto-oligosaccharides (GOS), fructo-oligosaccharides (FOS), lactulose, derivatives of galactose, and β-glucans [17] have been studied for their prebiotic capacity in IBD [18]. Prebiotics are non-digestible food components that selectively stimulate the growth or activity of desirable microorganisms, like *Bifidobacteria* spp. and *Lactobacilli* spp., and are hypothesized to positively impact the gut microbiome and alleviate IBD symptoms [17,19]. Despite these promising prospects, a well-powered placebo-controlled trial of FOS (15 g/day) showed no clinical effect in active CD patients [20]. However, a recent pilot trial evaluating oligofructose/inulin supplementation (15 g/day) in inactive CD patients and their healthy siblings [21] showed more pronounced effects in the healthy siblings, including decreased blood CD^3+^ T cells. Nonetheless, improved intestinal permeability was observed in the patients [21]. Furthermore, both patients and siblings showed increased levels of total *Bifidobacteria* spp. and *Bifidobacterium longum* [21]. In siblings, there were additional increases in *Bifidobacterium adolescentis* and *Roseburia* spp., which were not observed in the patients [21]. Conversely, a pilot trial evaluating different levels of carbohydrate-restrictive diets positively impacted symptoms and reduced inflammation in pediatric CD patients, with more restrictive diets showing a greater improvement in resolving inflammation [22]. Nevertheless, a specific carbohydrate diet was not more effective than the Mediterranean diet in achieving symptomatic remission, fecal calprotectin, and the CRP (C-reactive protein) response in adult CD patients [13]. In the case of UC, prebiotic GOS administration (2.8 g/day) did not result in improved clinical outcomes. However, it did normalize the gut microbiota in milder patients by increasing the levels of *Bifidobacterium* spp. and *Christensenellaceae* spp. [23]. Nevertheless, these findings require validation in randomized controlled trials.

Furthermore, FOS (2 g per kg/day) has been shown to alleviate the pathological immune response and prevent intestinal barrier impairment in an acute mouse model of colitis [24]. Additionally, various native starches derived from, e.g., potatoes, peas [25], and polysaccharides from the fruits of *Lycium barbarum* L. (200 mg/kg body weight/day) have been demonstrated to reduce disease activity and histologic damage in mouse models of colitis, as well as to support gut microbiota homeostasis by inhibiting rather pathogenic bacteria [26]. Pectic polysaccharides from guavira pomace, a co-product from the fruit pulp industry, have been shown to reverse increased permeability and transepithelial electrical resistance, potentially offering therapeutic benefits for treating abdominal pain and UC [27]. Furthermore, fruits like cranberries (30 g/day of freeze-dried cranberry powder) have been shown to attenuate changes in microbiota composition and functionality induced by an animal-based diet in healthy adults [28]. Glycolipids derived from tilapia heads have also been shown to reduce inflammation, similar to sulfasalazine controls, while regulating the gut microbiota composition by increasing beneficial bacteria and decreasing harmful ones [29].

Chitosan, an amino-polysaccharide derived from chitin, which can be obtained from the shells of crustaceans, fish scales, insects, and fungi, has attracted specific interest [30]. Its non-toxic nature, mucoadhesive properties, and ability to load and transport drugs make it an ideal biopolymer for developing transmucosal drug delivery systems [30]. Indeed, chitosan has been shown to have intestinal barrier-enhancing properties, as well as microbiota-modulatory properties, both in vivo (150 mg/kg) and in vitro [31,32]. Unlike commercially available gastro-resistant polymers, chitosan possesses the “generally regarded as safe” (GRAS) status, appointing it as a potentially safe option for long-term therapy in chronic diseases like IBD [33,34,35].

While dietary fiber is beneficial for those with normal microbial fermentative capacity, due to the production of short-chain fatty acids, certain patients with active IBD may experience negative effects from certain dietary fibers, due to decreased fermentative microbial activity [36]. The response to dietary fiber in IBD varies significantly and is influenced by individual differences in baseline gut microbiome composition and disease activity, which are shaped by dietary habits [20,37,38,39]. Therefore, a combination of dietary carbohydrates or prebiotics with probiotics (synbiotics) provides a potential solution to this issue [17,40].

## 4. Proteins

Historically, proteins have been evaluated primarily for their ability to supply essential amino acids and support protein synthesis [41]. Animal-derived proteins, particularly those found in red and processed meats, have been associated with increased inflammation and the exacerbation of IBD symptoms in animal models, whereas plant-derived proteins exhibit anti-inflammatory properties that can help mitigate disease activity [42,43]. In humans, a diet low in red and processed meat may reduce flare-ups of UC but has not shown effectiveness in preventing relapses of CD [44,45]. Other protein-rich components of the Mediterranean diet are dairy products, including yogurt and, increasingly, also kefir. The latter, being a fermented product rich in *Lactobacillus* spp., has especially gained increasing attention in IBD. In a rat model, a dose-dependent effect has been observed, where moderate kefir administration (PBS/10% kefir) provided better outcomes than higher doses (PBS/30% kefir) [46]. In humans, a randomized clinical trial found that kefir supplementation (400 mL/day) modulated the gut microbiota and improved short-term quality of life for IBD patients [47]. Probiotic Lactobacilli from kefir were shown to downregulate T cells in the inflammatory lamina propria of active IBD patients, suggesting that the microbial content of kefir may play a significant role [48]. However, animal-derived proteins from fish, poultry, and dairy products are essential components of the Mediterranean diet, providing necessary amino acids, vitamin B12, and heme iron, which are crucial for metabolic processes and maintaining muscle mass [15].

In addition to animal-derived proteins, plant-derived proteins are well-represented in the Mediterranean diet and are found in legumes, nuts, and seeds, which are consumed daily [15]. These plant proteins can significantly increase anti-inflammatory butyrate-producing bacteria, enhance bacterial diversity, and reduce pro-inflammatory bacteria [49]. Specifically, a meta-analysis identified high protein intake as a risk factor for IBD in an Asian population, potentially due to a dietary shift away from traditional plant-based patterns [43].

Certain amino acids, such as leucine, isoleucine, valine, glutamine, and glutamate (all at 3 g/L), have been selectively utilized in colonic microbiota studies in piglets, resulting in the production of health-promoting short-chain fatty acids (acetate and propionate), highlighting their prebiotic potential [50]. Specific amino acids, particularly tryptophan, are being intensively studied for their role in IBD pathophysiology [51]. Alterations in tryptophan metabolism have been linked to dysregulated immune responses in chronic inflammatory conditions like IBD [51]. Tryptophan can be converted into indole metabolites by the gut microbiota; these promote epithelial barrier functions and exert anti-inflammatory effects [52]. For example, a diet low in fat and high in fiber has been shown to be associated with a significant increase in tryptophan concentrations in UC patients [53]. Likewise, a pilot study demonstrated shifts in microbiota composition and increased tryptophan metabolites after four days of the Mediterranean diet [54]. Although the exact mechanisms remain unclear, it is hypothesized to be mediated by metabolic interactions in the gut microbiota [55]. In addition, both in vivo work and human data have indicated a role for tryptophan metabolite supplementation, as xanthurenic (400 mg/kg daily) and kynurenic acids (300 mg/kg daily) could decrease colitis severity [56]. Additionally, manipulating the endogenous tryptophan metabolism with recombinant aminoadipate aminotransferase showed protective effects [56]. However, a clinical trial on 5-hydroxytryptophan supplementation (50 to 3000 mg/day) showed no improvement in fatigue for patients in remission, indicating that further research is needed [57]. Targeting tryptophan metabolism remains a promising strategy for managing inflammation and restoring the immune balance in IBD patients.

## 5. Fats

High-fat diets, particularly those rich in saturated fats, such as the Western diet, have been linked to increased intestinal inflammation and the exacerbation of IBD symptoms in animal models by causing a dysbalanced gut microbiome, or dysbiosis and impaired intestinal barrier integrity [58,59]. In contrast, the Mediterranean diet is low in saturated fat, although higher in unsaturated fats, and a fat blend based on this diet was shown to protect against colitis development in mucin-2 (MUC2)-deficient mice [60]. Specific dietary interventions that are low in fat and high in fiber were shown to reduce levels of inflammatory markers and increase quality of life in UC [53], and to induce a rapid response in active pediatric CD patients [61]. These studies indicate a potential role for dietary fat in IBD, depending on its composition.

A major source of fat in the Mediterranean diet is olive oil [62,63], which has a high content of MUFA (65–83%), particularly oleic acid, along with some polyunsaturated fatty acids (PUFAs) such as linoleic acid. This lipid profile has been associated with protective effects against coronary, autoimmune, and inflammatory disorders [64]. Moreover, olive oil is considered to be rich in polyphenols, which contribute to its antioxidant and anti-inflammatory properties, further enhancing its potential health benefits. Several studies have investigated the supplementation of olive oil, of which an extensive overview is given by Vrdoljak et al. (2022) [62]. In summary, olive oil is an excellent dietary compound that may support traditional IBD drugs and assist patients in controlling their condition [62]. However, not all results are positive and seem to be dependent on variations in composition, especially with regard to polyphenol content (Table 1). For example, some researchers increased the polyphenolic content by using extra-virgin olive oil that was enhanced by its unsaponifiable fraction and enriched with specific polyphenols like hydroxytyrosol, which may have contributed to its effectiveness [62,65,66]. Of note, the polyphenol content in extra-virgin olive oil can vary significantly, depending on the olive cultivar, the abundance of secoiridoids and their derivatives, and the oil production and storage processes [67]. Indeed, a recent preclinical study investigated the effects of using extra virgin olive oils from four Italian cultivars known for their high polyphenolic content on mouse models. The study found that using these oils helped to reduce rectal bleeding and improve intestinal permeability. Additionally, the study observed a decrease in the expression of pro-inflammatory cytokines and an improvement in the histopathological characteristics of inflammation [68]. However, differences between cultivars were observed; therefore, a detailed characterization of monocultivar extra-virgin olive oils might be useful for a more targeted nutraceutical choice [68]. Additionally, oleuropein appears to be capable of downregulating inflammatory cytokines such as interleukin-6 (IL-6), metalloprotease secretion, and COX-2 [69]. More recently, protective effects were observed with synbiotic treatment in an in vivo UC model comprising *Lactobacillus plantarum SC-5* and tyrosol extracted from olive oil [70]. In addition, hydroxytyrosol was shown to counteract ERK1/2 and mTOR activation, pro-inflammatory cytokine release, and autophagy in primary human colonic cells, and was, therefore, hypothesized to be the most important anti-inflammatory and antioxidant compound present in olive oil [71]. Nevertheless, very limited human studies are available; thus, large, multicentric, randomized control trials evaluating the effects of olive oil in modulating the course of IBD are required.

Furthermore, certain fatty acids, such as omega-3 polyunsaturated fatty acids (PUFAs) originating from the consumption of fish [72] and from plant sources, including seeds, have been shown to possess anti-inflammatory properties; therefore, they may alleviate IBD symptoms by modulating immune responses and reducing pro-inflammatory cytokine production [73]. The rectal administration of fish oil (2 mL), known to be high in omega-3 PUFAs, has been shown to have beneficial effects that were mutualistic to mesalamine treatment [74]. Additionally, eicosapentaenoic acid (500 mg, twice daily) was shown to reduce fecal calprotectin and prevent relapses in UC [75]. This was confirmed by an evaluation of tuna oil in preclinical UC models [76]. Moreover, marine phospholipid nanoliposomes combining omega-3 PUFAs with phosphatidylcholine have been shown to be preferentially distributed in the inflamed gut and to delay disease onset upon prophylactic administration in an in vivo colitis model [77]. In humans, the effects of omega 3 on IBD are still uncertain. While some studies suggest the anti-inflammatory effects of omega-3 fatty acids in IBD [78,79], a recent meta-analysis of randomized controlled trials concluded that PUFA supplementation has little or no effect on the prevention or treatment of IBD [80]. Furthermore, it has been shown that an excess of PUFAs in the diet may even compromise gut health by disrupting epithelial stress responses [81]. Consequently, more studies are needed; in the meantime, the moderate supplementation of fish oil, algae, and their derivatives could be considered [73]. Additionally, nuts are often consumed in the Mediterranean diet and are an important source of sterols. Sterols have previously been shown to have anti-inflammatory and antioxidant properties, which may reduce inflammation in IBD patients [82,83,84]. For example, studies have demonstrated that in those with chronic inflammatory disorders, nut intake is linked to lower levels of inflammatory markers like CRP and IL-6 [85]. Additionally, the polyphenolic [86,87,88,89] (flavonoids) and phenolic acids [90,91,92,93] in nuts enhance their antioxidant activity, next to having barrier protection and microbiota modulatory potential.

Elsewhere, seafood and nuts have also been associated with IBD development in another case-control study [94]. Additionally, lower uptake of nuts, seeds, and yogurt was observed pre- and post-IBD diagnosis in a retrospective cohort study [95]. Therefore, understanding the intricate relationship between fat, its metabolism, and IBD in clinical and preclinical models is crucial for developing targeted dietary interventions and therapeutic strategies to effectively manage these chronic inflammatory disorders.

## 6. Red Wine

Red wine is moderately consumed in the Mediterranean diet and both clinical and preclinical studies have suggested that moderate consumption of red wine may confer protective benefits against the development and progression of IBD [96,97,98,99,100]. Importantly, some clinical studies have reported associations between moderate red wine consumption and a reduced risk of IBD incidence and severity, possibly attributable to its anti-inflammatory and antioxidant effects [101]. The first compound of red wine that has previously been studied is resveratrol. Preclinical studies have demonstrated that resveratrol (1 mg/kg/day) may possess anti-inflammatory properties, modulate immune responses, and reduce oxidative stress, and would thus be able to attenuate intestinal inflammation [96,97]. In addition, resveratrol administration led to increased levels of *Lactobacilli* and *Bifidobacteria*, as well as decreased levels of *Enterobacteria* in a disease model [97]. A second compound of interest is tannin, which is highly present in red wine. Limited clinical research has been performed in the framework of IBD, although tannins have been studied in relation to the progression of dextran sodium sulfate (DSS)-induced colitis in a preclinical setting [98]. In these experiments, a correlation between the quantity of tannins (0, 10, 50, and 250 mg/kg) and the outcomes of DSS-induced colitis was observed [98]. In addition, their effects were shown to be mediated by tannin-induced enrichment of the microbial metabolite, gallic acid [98]. Although resveratrol and tannins might have beneficial effects, it is essential to note that excessive alcohol consumption, including red wine, may exacerbate IBD symptoms and increase the disease severity [101]. Furthermore, it is uncertain whether these positive effects can be achieved by drinking wine alone, as the beverage only contains limited concentrations of these compounds [102]. As a result, further research is required to elucidate the precise mechanisms and to establish optimal and potential health-benefitting consumption patterns.

## 7. Other Specific Nutrients and Vitamins

Consumption of a wide variety of plant-derived components in the Mediterranean diet promotes exposure to bioactive molecules that can modify inflammation (Table 2).

Quercetin is a plant pigment and a potent antioxidative flavonoid that is found mostly in onions, grapes, berries, cherries, broccoli, and citrus fruits [103]. Quercetin intake in a typical Mediterranean diet may vary, but estimates suggest that it could range from 10 to 20 mg per day from foods alone [104]. It is a versatile antioxidant that has been shown to possess protective abilities against the tissue injury induced by various drug toxicities, together with anti-cancer, anti-ulcer, anti-diabetic, and anti-hypertensive properties [105]. From animal studies, it emerges that quercetin (100–1500 ppm in diet) helps to protect the enterocytes from apoptosis caused by oxidative stress, which, in turn, helps provide protection against colitis [106,107]. Moreover, it increases intestinal microbial diversity by promoting the growth of *Bacteroides* spp., *Bifidobacterium* spp., *Lactobacillus* spp., and *Clostridium* spp., while reducing the levels of *Fusobacterium* spp. and *Enterococcus* spp. [108]. Additionally, quercetin seems to reduce intestinal permeability by enhancing the expression of tight junctions [107,109]. It also promotes the proliferation of intestinal cells and supports the regenerative capacity of intestinal mesenchymal stem cells from animals [107,109]. In addition, this component also intervenes in the regulation of the immune system, reducing the infiltration of neutrophils and macrophages into the colonic tissue of mice. Furthermore, it inhibits chronic intestinal inflammation by reducing macrophage infiltration by several pro-inflammatory cytokines, such as TNF-α, IL-1β, IL-6, and IL-17. Finally, it promotes anti-inflammatory IL-10 secretion in the colonic tissues [110]. In summary, quercetin might be useful in IBD management [111,112]. However, its low water solubility and bioavailability have been a significant challenge [113]. To address this issue, various strategies are actively being investigated to improve its absorption. For example, recent studies have demonstrated remarkable success in using quercetin nanoparticles [114,115,116]. These nanoparticles have shown regulatory effects on the gut microbiota and their metabolites like SCFAs, resulting in a significant reduction of inflammatory infiltration of the colon [114,115,116].

**Table 2:** Overview of the discussed nutrients or compounds, their subtype, and in which foods they are present.

Nutrient/Compound	Subtype	Found in
Quercetin	Pigment	Onions, grapes, berries, cherries, broccoli, and citrus fruits [103]
Astaxanthin	Pigment	Microalgae, salmon, shrimp, and krill [117]
Lycopene	Carotenoid	Tomatoes and other red fruits [118]
Curcumin	Spice	Turmeric [118]
Epigallocatechin gallate	Polyphenol	Green tea [119]
Vitamin B9 or folate	Vitamin	Dark green leafy vegetables like lettuce, asparagus, broccoli, beans, peanuts, sunflower seeds, fresh fruits, whole grains, liver, eggs, fish, and seafood [120]
Vitamin D or calciferol	Vitamin	Cheese, beef liver, egg yolk, tuna, mackerel, and salmon [121]

Another pigment, astaxanthin, is a potent antioxidant belonging to the carotenoid family. It is primarily sourced from microalgae, salmon, shrimp, and krill, and possesses remarkable anti-inflammatory properties attributable to its ability to scavenge free radicals, inhibit oxidative stress, and modulate immune responses [117]. The Mediterranean diet encourages the consumption of about three servings of fish or seafood per week, which could translate into an estimated intake of 1–5 mg of astaxanthin per week [122]. In preclinical in vivo studies [123,124,125,126,127,128,129], astaxanthin has demonstrated its efficacy in attenuating inflammation, preserving mucosal integrity, and regulating the expression of pro-inflammatory cytokines and mediators implicated in IBD pathogenesis. For example, the decreased expression of IL-1β, IL-6, TNF-α, IL-36α, and IL-36γ has been observed after treatment with 4 mg astaxanthin in nanocarriers [123]. Clinical investigations evaluating astaxanthin supplementation in other diseases like type 2 diabetes, arthritis, and endometriosis have shown promising results in modulating the observed inflammation and reducing disease severity [130,131,132]. Although further research in the framework of IBD is still required, these findings may support the potential application of diets rich in astaxanthin, like the Mediterranean diet.

The carotenoid lycopene, which is mostly present in tomatoes and other red fruits, has previously been shown to be involved in reducing inflammation [118]. Lycopene may scavenge reactive oxygen species, suppress pro-inflammatory cytokines, and modify the signaling pathways linked to immune responses [118]. Furthermore, lycopene shows promise in lowering oxidative stress and maintaining intestinal barrier integrity [118], two important aspects of IBD pathogenesis and the reason it gained interest as a dietary supplement. The average dose of lycopene in the Mediterranean diet is difficult to quantify, considering the high variability of plant products; however, based on typical daily consumption patterns, the Mediterranean diet may provide approximately 6–15 mg of lycopene [133]. Research employing animal models has shown positive findings regarding lycopene’s (12 mg/kg) ability to reduce inflammation and disease severity by improving epithelial barrier functions and inhibiting *E. coli* adhesion [134,135]. However, further research is mandatory; Mendelian randomization studies have suggested that certain circulating antioxidants, minerals, and vitamins might also be associated with IBD development [136].

Although not part of the traditional Mediterranean diet, turmeric’s bioactive ingredient, curcumin, is increasingly used in the modern Mediterranean kitchen and has gained interest due to its possible beneficial effects for different diseases, including IBD [137]. Curcumin’s anti-inflammatory effects are mediated via a number of pathways, including immune cell function regulation, the inhibition of inflammatory cytokines, and suppression of the NF-κB signaling pathway [138,139]. Furthermore, curcumin possesses antioxidant properties by scavenging free radicals and lowering oxidative stress—two important aspects of the pathophysiology of IBD [138]. Preclinical research employing animal models of IBD has shown encouraging findings, suggesting that curcumin supplementation (100 mg/kg) may alleviate colitis and improve dysbiosis [137,140]. Curcumin’s clinical translation is hampered by certain issues, including limited bioavailability and fast metabolism, despite its promising medicinal promise [141]. Several strategies, including combination therapy and nano-formulations, are being investigated to improve curcumin’s bioavailability and effectiveness for improved IBD management [141]. For example, a pilot trial showed positive results, including clinical and endoscopic efficacy and safety for IBD, when assessing Theracurmin^®^, a nanoparticle-based drug delivery system with increased bioavailability [141]. However, larger studies are required to confirm these findings.

Green tea is often added to the Mediterranean diet and contains high levels of the polyphenol epigallocatechin gallate (ECGC) [119]. ECGC has been shown to be able to improve lipid metabolism and to reduce inflammation and oxidative stress in an in vivo model of obesity [119]. In addition, decreased inflammation has also been observed in multiple sclerosis and type 2 diabetes upon supplementation with ECGC [142,143]. Nevertheless, these effects remain to be evaluated in IBD.

Vitamin D or calciferol deficiency is a common issue in IBD due to several reasons, such as immunosuppressive treatments affecting sun exposure, dietary restrictions, and impaired absorption [8,144]. Besides playing a critical role in bone and muscle health, vitamin D also supports the integrity of the gut epithelium, innate immune barrier function, and eubiosis [144,145]. Vitamin D intake was positively associated with greater adherence to the Mediterranean diet [146]. This result may seem surprising, given that vitamin D-rich foods, like cheese, beef liver, egg yolk, tuna, mackerel, and salmon, are not typical compounds of the Mediterranean diet per se, except for some specific cheeses and fish [147]. Indeed, the Mediterranean diet does not inherently provide high levels of vitamin D [121]. However, this diet enhances the body’s ability to absorb vitamin D effectively, which could be mediated by the gut microbiome [121]. This has been confirmed in a cross-sectional study in which adherence to the Mediterranean diet for a year could increase vitamin D levels [148]. Specifically, a Mediterranean diet intervention appeared to have a greater impact on the bacterial populations of the main phyla of Firmicutes and Proteobacteria, with Firmicutes increasing and Proteobacteria decreasing [148]. Unfortunately, the majority of clinical intervention studies supplementing vitamin D have failed [149], indicating that there might be other interactions of importance; therefore, further research is required.

## 8. Multiple Nutrients United in One Diet

A Mediterranean dietary pattern is rich in various potential beneficial compounds, which collectively exert anti-inflammatory, antioxidant, and gut microbiota-modulating effects in preclinical research [9] and are discussed throughout this review. Nevertheless, it has to be noted that understanding the role of a specific class of compounds in foods that promote health is challenging, due to chemically complex food matrices. Often, the individual compounds tested in clinical trials lack sufficient effectiveness or yield negative results. This could potentially be explained by the lack of preclinical dose optimization and potential discrepancies between the evaluated doses and their actual presence in the Mediterranean diet, leading to over- or underdosing. Nevertheless, all these compounds are united in the Mediterranean diet, in which the different nutrients might exert synergistic effects. Therefore, several clinical studies have been performed, aiming to evaluate the effects of this diet in clinical trials, which have extensively been reviewed by Godny and Dotan (2024) [9]. In summary, implementation of the Mediterranean diet has been associated with beneficial results in IBD, especially in contrast to Western diets. Epidemiological evidence suggests a potential association between the Mediterranean diet and reduced CD risk. Furthermore, adherence to this diet has shown moderate improvements in clinical and inflammatory markers among active CD patients and has helped maintain low fecal calprotectin levels in patients with quiescent UC [13]. Mechanistically, the Mediterranean diet fosters the proliferation of beneficial gut bacteria and enhances microbial community diversity.

## 9. Concluding Discussion and Future Perspectives

**Figure 1:**
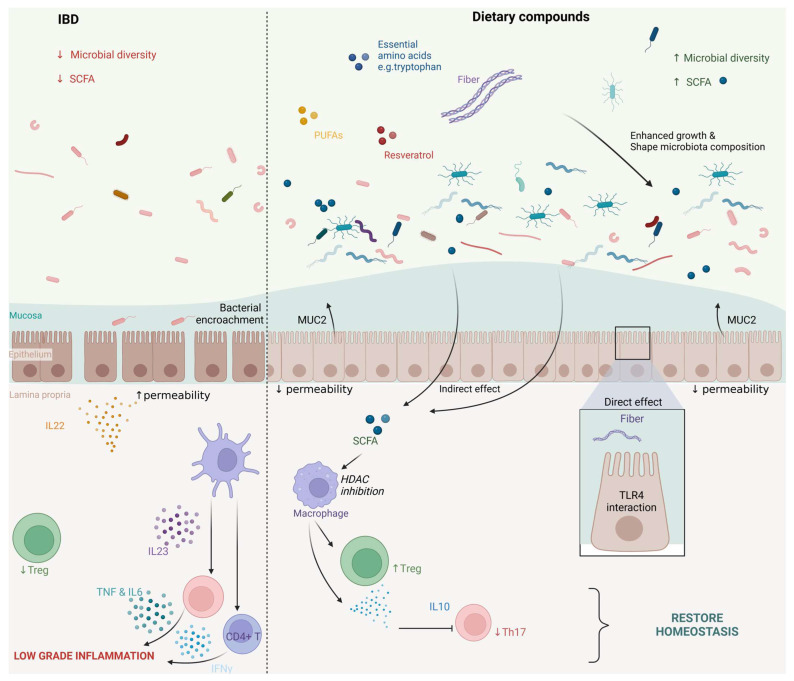
Overview of the potential mechanisms of the nutrients present in the Mediterranean diet, including fiber, essential amino acids such as tryptophan, polyunsaturated fatty acids, and resveratrol. SCFA: short-chain fatty acids; HDAC: histone deacetylation.

Adherence to the Mediterranean diet may offer a multifaceted approach to managing inflammation in IBD through its diverse array of beneficial compounds, not only by shaping the body’s microbial community but also by changing the micro-environment. Therefore, recently published American guidelines suggest that all patients in remission or with mild to moderate symptoms should be advised to follow a Mediterranean diet unless there would be any contraindication [8]. Moreover, additives such as emulsifiers are relatively limited in abundance in the Mediterranean diet, which, in turn, is beneficial since emulsifiers have been associated with IBD progression [12,150]. Thus, along with many potentially beneficial compounds and nutrients, the Mediterranean diet contains limited amounts of harmful ones. Overall, strong clinical evidence provided by randomized controlled trials is lacking, and there is also a scarcity of information on the small intestines. Moreover, although the observed preclinical effects might be influenced by changes in microbial composition, these changes may subsequently exert indirect effects via SCFA by the inhibition of histone deacetylation (HDAC) [151] (Figure 1). The precise mechanisms for most nutrients remain to be elucidated. To bridge these gaps, it is essential to conduct further research to understand the interactions between nutrients and the microbiome in both the small and large intestines, paving the way for more effective clinical interventions.

Nevertheless, specific dietary compounds such as chitosan could be used for the encapsulation of drugs [31,35], which might serve as an add-on, for example, for probiotic or oral fecal microbiota delivery, in order to increase its efficacy [35,152,153]. Moreover, there might be a synergistic effect of IBD therapies and adherence to the Mediterranean diet, which could break the currently observed therapeutic ceiling. Although not part of the daily IBD clinical recommendations, several diets have been evaluated in the framework of fecal microbiota transplantation (FMT). For example, an exclusion diet alone achieved higher remission rates and mucosal healing compared to a single dose of FMT, with or without a supportive exclusion diet [6]. Additionally, seven weekly FMT sessions, supported with an anti-inflammatory diet, were shown to effectively induce and maintain deep remission compared to standard medical therapy [5], which highlights the potential of diet as an add-on strategy in IBD. Contrastingly, diets low in fiber [154], like exclusive enteral nutrition (EEN), have been shown to be efficacious in inducing clinical remission in CD, albeit with stronger evidence in children [8], and are hypothesized to change the microbial composition [155]. Although this change is not always observed as being healthier, it may disrupt the established dysbiotic microbial communities, thereby allowing for the re-colonization and establishment of communities that promote a more balanced interaction with the host. In this regard, a Mediterranean lifestyle might be beneficial as a maintenance approach. However, more research is required to evaluate the interactions of a Mediterranean diet or lifestyle and treatment options for the management of IBD.

**Figure 2:**
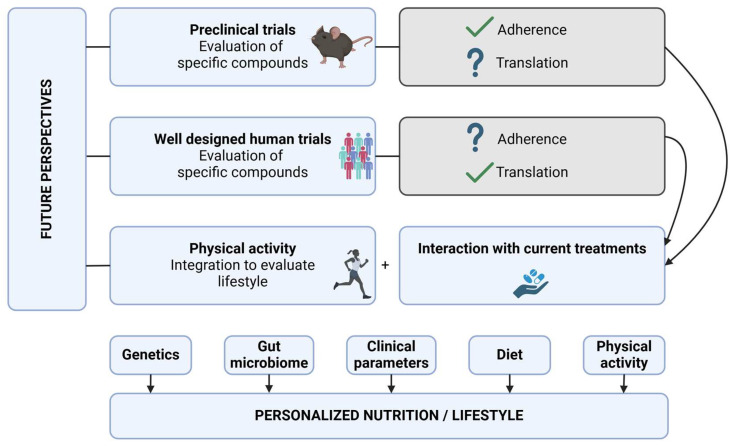
Overview of future perspectives. A major issue associated with dietary interventions is adherence to the diet. Therefore, preclinical in vivo trials can aid in evaluating the effects of specific diets and nutrients. Other approaches include well-designed human trials in which diets and nutrients are evaluated, as well as capsules that can be derived from and/or contain beneficial or existing compounds. In addition, the interaction of dietary interventions or nutrients with existing treatments and those in development should be investigated. Finally, the effects of physical activity should also be integrated in future work. The integration of genetic factors, the gut microbiome, clinical parameters, diet, and physical activity using machine learning techniques will lead to personalized nutrition or lifestyle advice in both health and disease.

A major problem in human dietary research is the evaluation of adherence to a specific diet, due to a multitude of available scales, as well as the cumbersome implementation of a true control group, which leads to very heterogeneous results [156] and is, therefore, concerning. While the Mediterranean diet offers potential benefits for IBD patients and is relatively less restrictive than other diets, adherence might remain a challenge due to gastrointestinal symptoms [9], psychological factors, and a lack of tailored support. Addressing these barriers through education, individualized dietary plans, and supportive interventions is crucial for enhancing adherence and ultimately improving health outcomes for IBD patients. Hence, animal models are perfectly suited for performing dietary intervention studies (Figure 2). In this regard, many in vivo studies have been performed involving Western and regular chow in mice; however, research investigating the Mediterranean diet is rather limited in availability. Nevertheless, to avoid results getting lost in translation from obtained in vivo data to real-world patient information, dietary effects could be evaluated in patient-derived ex vivo models, such as biopsies [157] and organoid-derived monolayer cultures [158]; humanized in vivo models [159] or even studies with healthy individuals could be considered for evaluating specific compounds, prior to progressing to trials involving patients. Further research could include diets versus single food-component supplementation to evaluate their specific effects. Specifically, in clinical trials, some foods might be high in a specific compound, but, for standardization purposes, it might be favorable to use a prebiotic compound.

Beyond dietary composition, the Mediterranean lifestyle is characterized by communal meals, leisurely dining, and physical activity. This active lifestyle has been associated with a reduced risk of chronic diseases such as cardiovascular ailments, metabolic disorders, and certain cancers, as well as improved longevity and overall well-being. A recent clinical trial in pediatric IBD patients has shown that a 12-week lifestyle intervention including physical activity improved fatigue perception, quality of life, and bowel symptoms [160]. Moreover, a multicenter cross-sectional study identified that IBD and its symptoms severely affect physical activity levels, due to fatigue and the fear of diarrhea [161]. In addition, physicians reported a lack of knowledge in this field; therefore, work remains to achieve optimization. However, more research is still required to evaluate the effects of physical activity on disease-specific quality of life, biomarkers, and general health status. Overall, applying the Mediterranean lifestyle in IBD serves as a model for promoting health and longevity through a balanced integration of dietary, social, and physical activities, thereby meriting a multidisciplinary management approach.

The Mediterranean lifestyle, especially its diet, is commonly embraced for its health benefits, but these effects may not be suitable for all IBD patients due to the highly variable course of the disease. Given that IBD patients frequently experience dysbiosis [2], and the Mediterranean diet’s benefits are often mediated through changes in the gut microbiota with a certain baseline presence, a one-size-fits-all approach is inadequate. Specifically, personalized dietary strategies (Figure 2) will be essential in the future [161]. This approach entails a multi-disciplinary evaluation of patients’ genetics, the unique gut microbiome, disease variability, physical activity, and other environmental factors, combined via machine learning techniques to personalize dietary interventions. These interventions aim to treat inflammation, symptoms, and dysbiosis. It is of the utmost importance to identify the effects of food compounds on both the host and microbiome to develop effective treatments. Investigating the complex interactions between lifestyle, nutrients, and IBD will provide an individualized yet multidisciplinary strategy for disease management. Therefore, further research is mandatory, including preclinical work to identify potential beneficial nutrients and optimal doses, followed by robust clinical trials.

## Figures and Tables

**Table 1:** Use of extra-virgin olive oil or its components in preclinical IBD models over the past five years.

Author (Year)	Disease	Model	Design	Ingredient	Outcomes
Hassan Motawea et al. (2020) [69]	UC	In vivo—Mice	Prospective interventional study	Oleuropein (350 mg/kg/day)	Reduction in: -mortality -DAI -oxidative stress -inflammation
Cariello et al. (2020) [68]	UC	In vivo—Mice	Prospective interventional study	High polyphenolic content EVO oil (dose NA)	Reduction in: -rectal bleeding -IL-1β, TGFβ, IL-6 -intestinal permeability
Yu et al. (2024) [70]	UC	In vivo—Mice	Prospective interventional study	Tyrosol (20 mg/kg/day)	Remission of colitis Improvement of inflammation
Santarelli et al. (2022) [71]	NA	In vitro—Primary Human Colonic Epithelial Cells	NA	Hydroxytyrosol (1 µM)	Reduction in: -cytokines and chemokines -tumorigenesis promotion

UC: ulcerative colitis; DAI: disease activity index; EVO: Extra Virgin olive; NA: Not applicable.
